# Supplementary material for: Evaluation of the Enzyme Inhibitory and Antioxidant Activities of Entada spiralis Stem Bark and Isolation of the Active Constituents
Source: Molecules. 2019 Mar 13;24(6):1006. doi: 10.3390/molecules24061006 (PMC6471705; doi:10.3390/molecules24061006)
Supplement: Supplementary file 1 [file molecules-24-01006-s001.pdf]

Evaluation of the Enzyme Inhibitory and Antioxidant Activities of *Entada spiralis* Stem Bark and Isolation of the Active Constituents

Fatimah Opeyemi Roheem [1], Siti Zaiton Mat Soad [1,*], Qamar Uddin Ahmed [1], Syed Adnan Ali Shah [2,3], Jalifah Latip [4] and Zainul Amiruddin Zakaria [5,6,*]

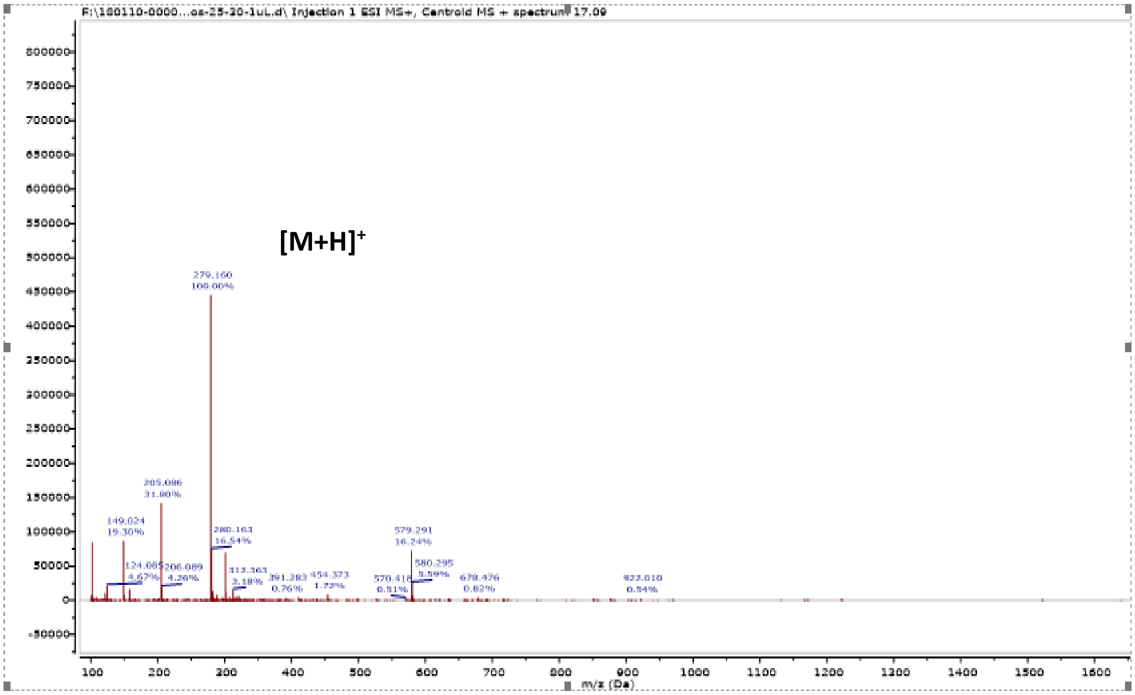

Fi

Figure S1. Mass spectrum of **FEQ-2**

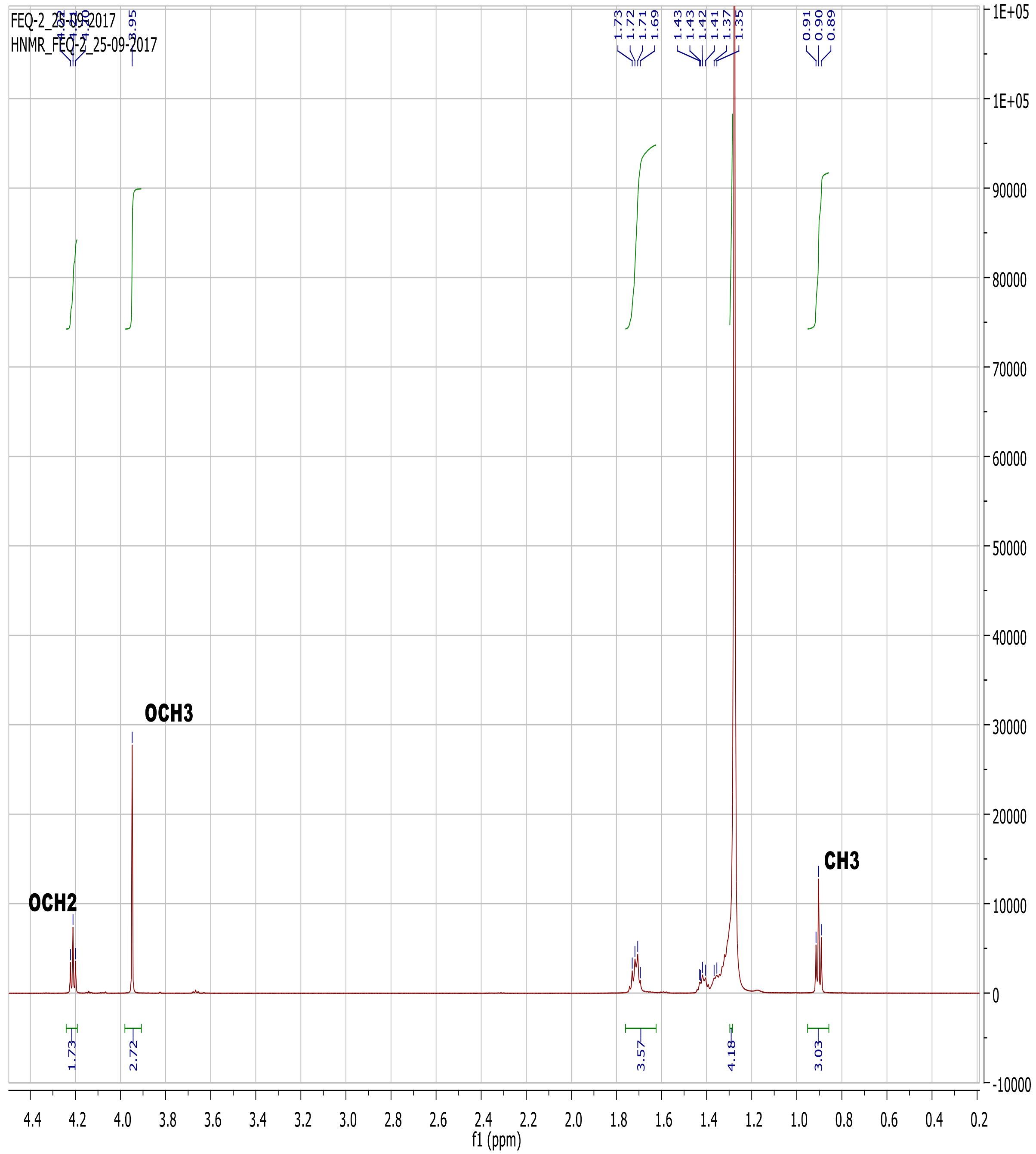

Figure S2(i). ^{1}H- NMR spectrum of **FEQ-2** (0.600- 4.400 PPM in $CDCl_3$)

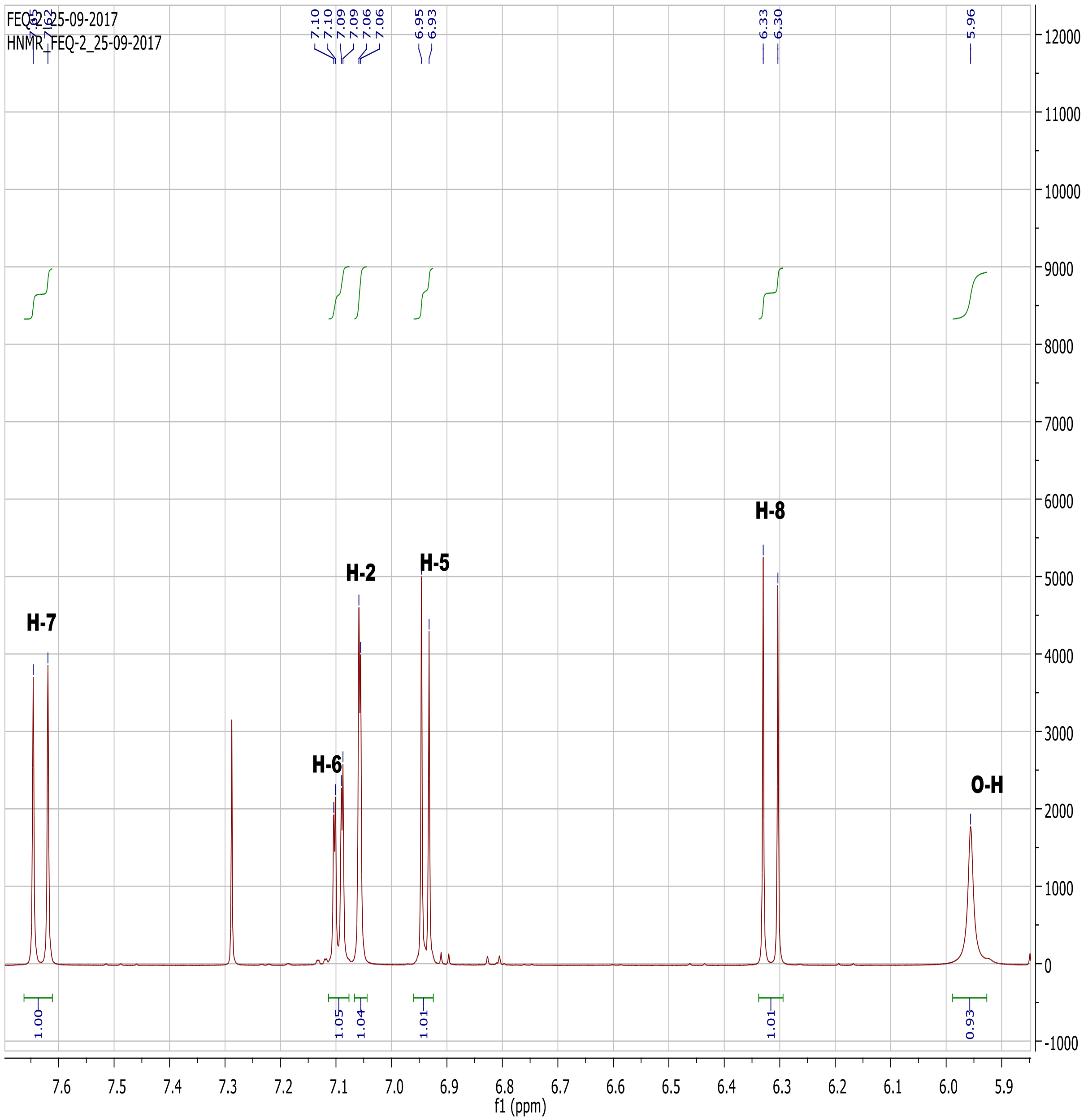

Figure S2 (ii). ^{1}H- NMR spectrum of **FEQ-2** (3.800- 7.800 PPM in $CDCl_3$)

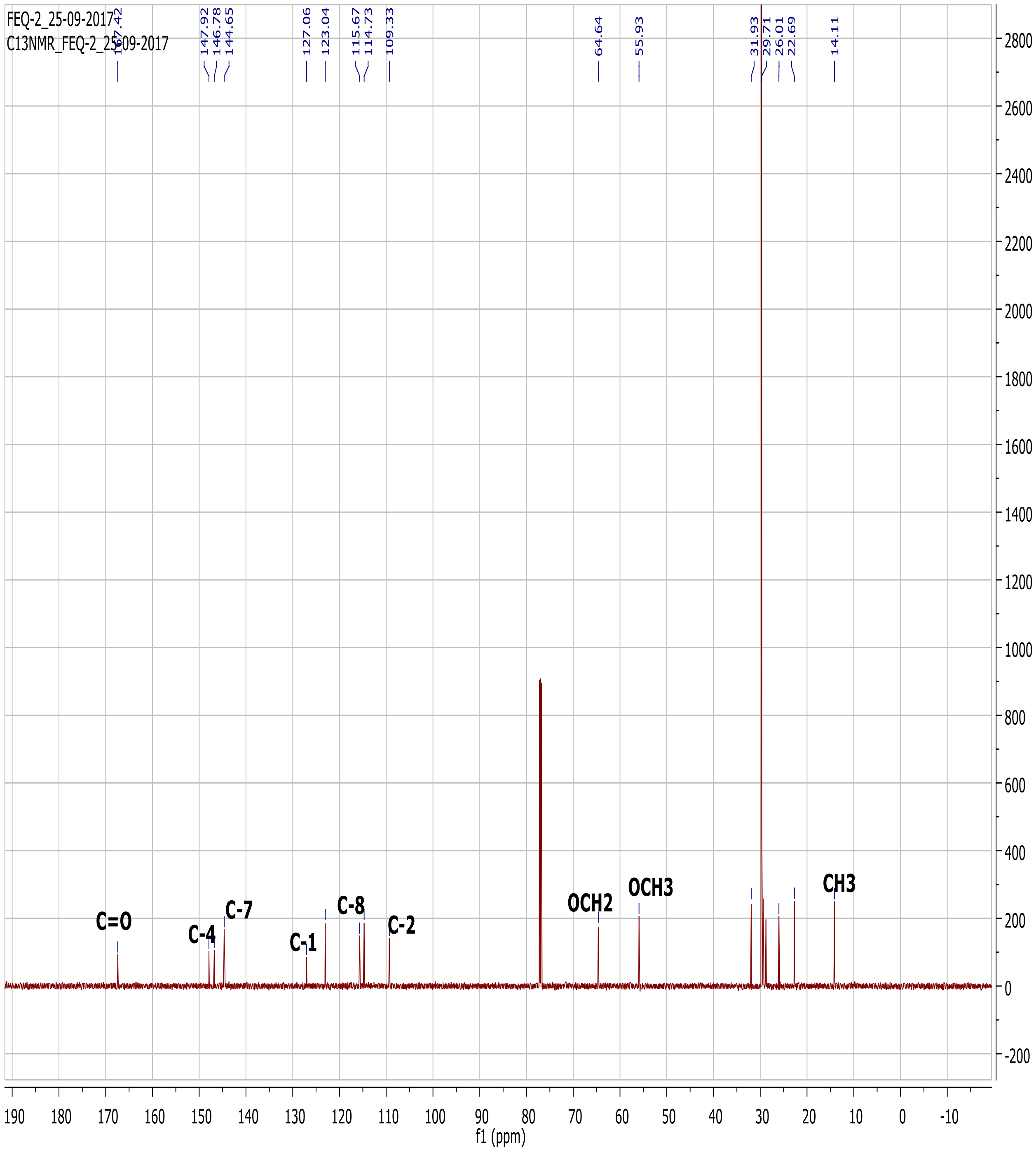

Figure S3. ^{13}C-NMR spectrum of **FEQ-2**

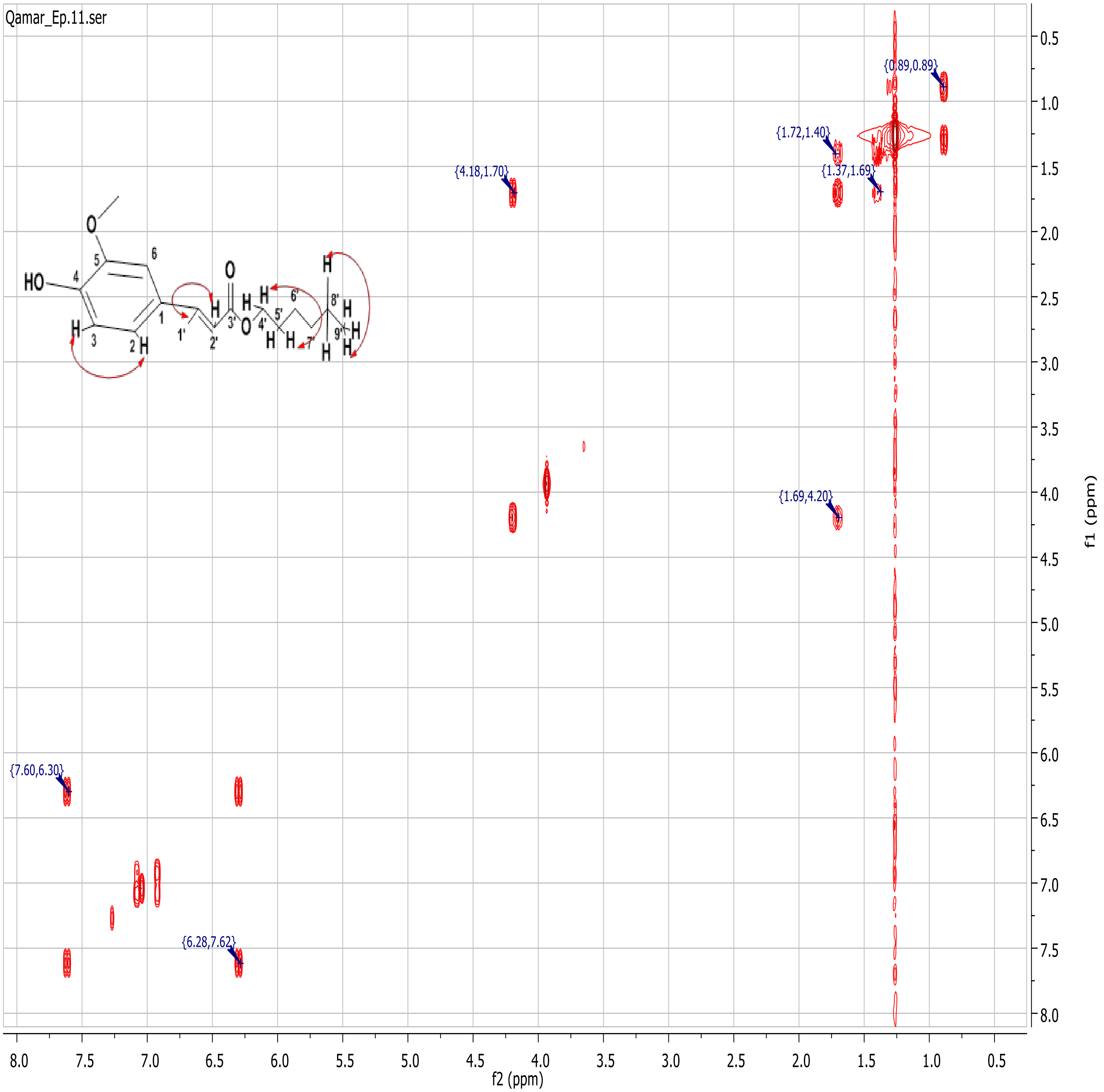

Figure S4. COSY spectrum of FEQ-2

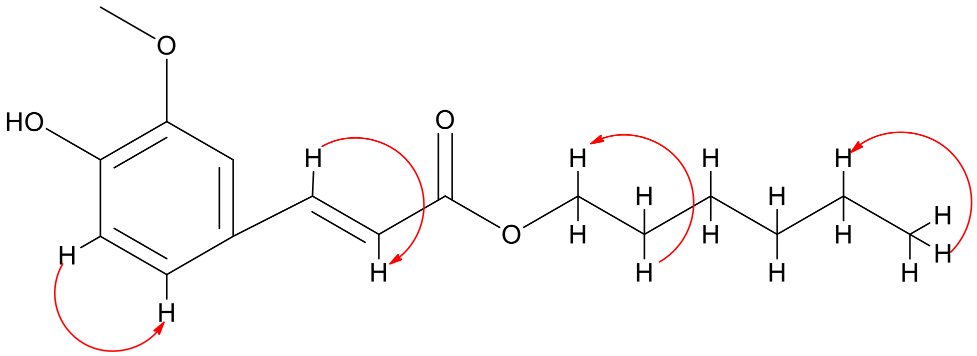

Figure 5. Significant ^{1}H-^{1}H COSY correlations for **FEQ-2** isolated from the methanol extract of *E. spiralis* stem bark.

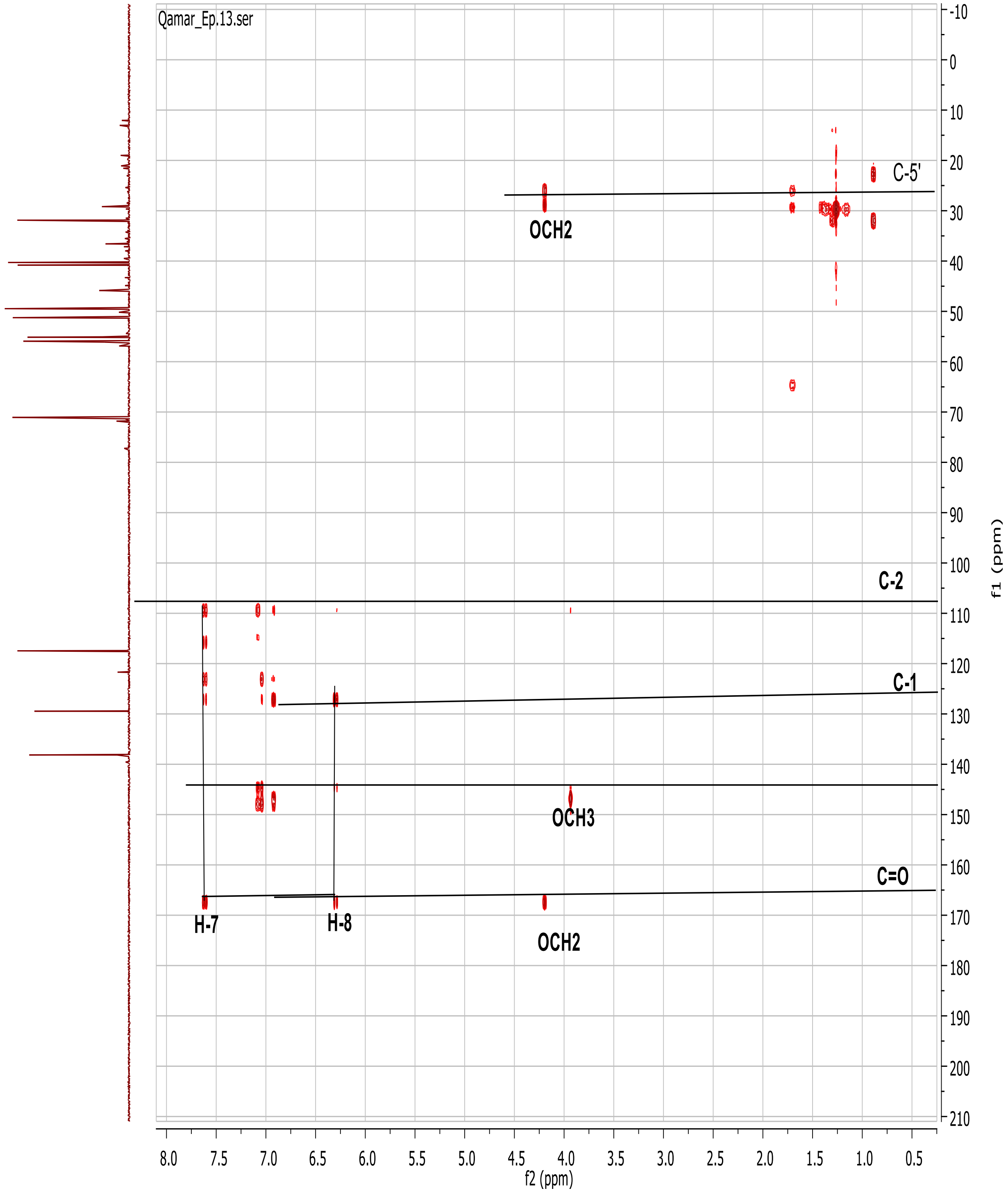

Figure S6. HMBC spectrum of **FEQ-2**

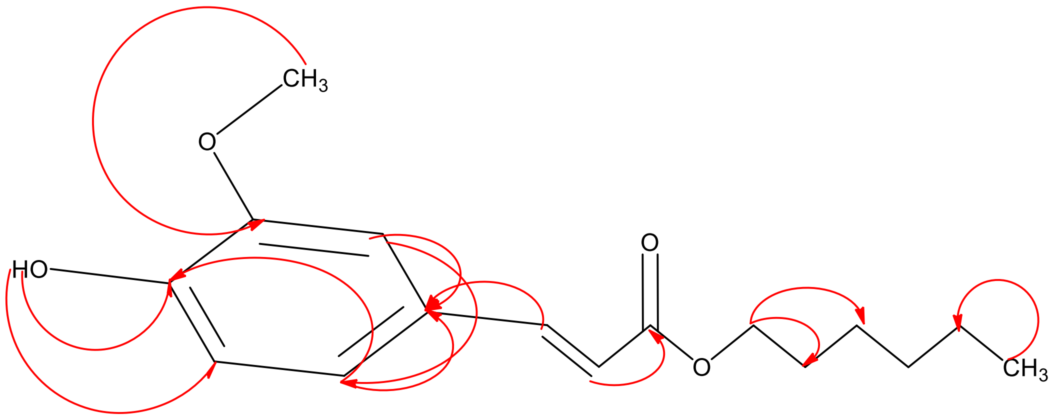

Figure S7. Significant HMBC correlations for **FEQ-2** isolated from the methanol extract of *E. spiralis* stem bark.
